# Optimization and Evaluation of Complementary Degrader Discovery Assays for Application in Screening

**DOI:** 10.1021/acsptsci.5c00195

**Published:** 2025-07-20

**Authors:** Johanna Huchting, Arjen Weller, Moyra Schweizer, Mona Brandt, Jan Heering, Maria Kuzikov, Markus Wolf, Jeanette Reinshagen, Markus A. Queisser, Philip Gribbon, Andrea Zaliani, Ole Pless, Aimo Kannt

**Affiliations:** † 587462 Fraunhofer Institute for Translational Medicine and Pharmacology ITMP, Schnackenburgallee 114, 22525 Hamburg, Germany; ‡ Medicines Research Centre, GSK, SG1 2NY Stevenage, U.K.; § Fraunhofer Institute for Translational Medicine and Pharmacology ITMP and Innovation Centre TheraNova, Theodor-Stern-Kai 7, 60596 Frankfurt am Main, Germany; ∥ Institute of Clinical Pharmacology, Goethe University Frankfurt, Theodor-Stern-Kai 7, 60596 Frankfurt am Main, Germany

**Keywords:** targeted protein degradation, molecular glue degrader, screening, assay validation, immunoassay, signal rescue

## Abstract

Targeted protein degradation (TPD) mediated by molecular glues is an innovative pharmaceutical paradigm. By binding to and modulating the surface of an E3-ligase component, molecular glue degraders can facilitate the recruitment of a specific target protein (or vice versa) and ultimately invoke target degradation. This mode of action results in specific challenges for the development of rational discovery strategies, and complex hit validation workflows may be required to reliably eliminate compounds that elicit nonspecific effects. With the aim to guide screening efforts, we optimized two orthogonal cell-based, target-centric assays for degrader discovery: (1) a time-resolved FRET assay directly quantifying the levels of a target protein and its degradation (signal inhibition) and (2) an assay coupling TPD to cell growth (signal rescue). To enable a deeper understanding of the individual assays’ strengths and limitations, we compared their statistical performance as well as respective hit populations by screening a specifically designed collection of about 1000 compounds containing well-annotated reference compounds and known frequent hitters (FHs). We found that the signal rescue format reliably and specifically captured active target degraders while efficiently filtering out interfering or FH compounds. Importantly, this format achieved to retrieve lower potency hits, which might be desirable in order to confidently include as many diverse chemical starting points as possible at the start of a drug discovery project.

## Graphical abstract

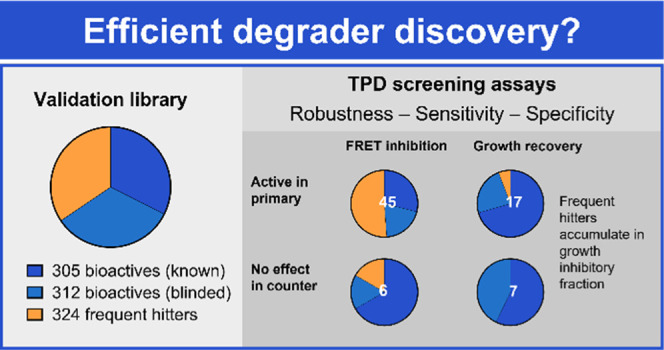

Traditionally, small-molecule therapeutics subsume modulators of protein function, such as inhibitors or activators. An increasingly important class of small molecules induces proximity between biological macromolecular entities, one being the biological target (e.g., a disease-modifying protein) and the other a biological effector. Molecular degraders, the most prominent type of proximity-based modalities, involve E3 ubiquitin ligases as biological effectors. They thus induce target protein (poly)­ubiquitylation and subsequent proteasomal degradation, thereby depleting the protein of interest. Typically, the molecular degrader is retained and can thus induce degradation of the next target protein molecule, resulting in a catalytic mode of action.^1^ While some molecular degraders are already in clinical use, with the molecular glue degrader (MGD) Lenalidomide (Revlimid) as the most prominent example, they have so far only retrospectively been shown to act via targeted protein degradation (TPD). Prospectively developed, advanced clinical candidates in this field largely belong to the heterobifunctional group of molecular degraders, such as proteolysis-targeting chimeras (PROTACs), that connect binders for target and E3-ligase via a linker.^2^ MGDs can reach proteins that lack classical ligandable allosteric or orthosteric pockets, such as transcription factors, and are similar to conventional small-molecule drugs in terms of classical drug-likeness. Systematic strategies for the prospective MGD discovery, however, are only beginning to emerge, and rational design principles are not fully understood.^3–5^

When screening for molecular degraders with target protein abundance as the primary readout, the presence of a target degrader results in a lower signal compared to vehicle control (signal inhibition), as would also compounds exhibiting rather unspecific effects such as cytotoxic compounds, translation inhibitors, etc. Consequently, stringent and extensive hit deconvolution cascades are needed to identify bona fide degraders from all hits of a screening library, and especially low-potency MGDs may be missed.^6^ In contrast to classical affinity-based modalities that follow an occupancy-driven mechanism, direct biophysical hit validation for catalytically acting degraders may be restricted. Functional degrader validation generally includes recovery of a target protein by proteasome inhibition or pharmacological inhibition of NEDDylation.^6^ However, in a recent work, Schwalm et al. have clearly demonstrated confounding effects of these strategies.^7^ Moreover, Vetma et al. have recently shown that cytotoxic compounds can disguise as targeted degraders even through profiling efforts, especially in the context of short-lived proteins.^8^

Our study compares target-centric, orthogonal assay formats for degrader discovery. It incorporates (1) direct measurement of target protein abundance (signal inhibition) as well as (2) an approach that couples TPD to a positive readout, specifically cell growth recovery (signal rescue). The latter format was first introduced to TPD by Koduri et al. and employs exogenous expression of a target-suicide kinase fusion protein, a strategy borrowed from cell fate control gene therapy.^9^ We rigorously address challenges in assay sensitivity and robustness and provide detailed guidance on assay implementation. With the aim of understanding the individual strengths and limitations of the different formats, we provide an in-depth analysis of screening results making use of a specifically designed validation library. This library leverages prior knowledge of the compounds’ biological effects as well as (predicted) compound promiscuity,^10^ i.e., frequently observed activities in a range of biological contexts. Such promiscuity may result from polypharmacology of a compound or pharmacological activity at central nodes in cellular pathways, leading to more generalized downstream effects (e.g., translation inhibitors).^11^ Hence, our setup rigorously challenges the assay formats in terms of specificity and enables a concise interpretation of the screening results. The findings from our study may guide TPD researchers during the setup and rigorous vetting of assays for molecular degrader discovery as well as provide orientation for designing an efficient cellular screening architecture for the detection of molecular degraders.

## Results and Discussion

### Direct End-Point Measurement of the Target Protein Level: Assay Adaptation

We implemented a homogeneous time-resolved FRET (HTRF) sandwich immunoassay where, after treatment with a potential degrader, cells are lysed and a FRET-labeled antibody pair is added to detect separate epitopes on the same target protein. The assay does not require any washing after cell lysis, and the read FRET signal is directly proportional to the amount of target protein in the sample. As a target, we selected the transcription factor IKZF1 (IKAROS family zinc finger 1). IKZF1 is targeted by numerous thoroughly characterized MGDs that utilize the E3 ligase component cereblon (CRBN) and are therefore being referred to as cereblon-E3-ligase-modulating drugs (CELMoDs).^12–20^ ^(review)^ A central aim of this study was a direct comparison of this signal inhibition assay to an orthogonal, signal rescue-type format, and hence, the choice of cell line had to consider the needs of both assays. For the signal rescue format, the assay principle relies on the expression of a target-suicide kinase fusion protein; consequently, we adapted and optimized both assay formats using the same genetically modified cell line. Following assay miniaturization to a 384-well format and automation, and with our focus set on achieving a robust setup suitable for screening, we then aimed to maximize the assay window (directly depending on maximal degradation %, *D*_max_) and minimize variation. For this, a 20 h treatment with pomalidomide seems best suited (Figure 1A), while for other target/degrader pairs, a 6 h treatment may already suffice (as we found for RBM39/indisulam, data not shown). Relative IKZF1 protein levels measured after 20 h of treatment with diverse CELMODs reflected well the published activities of these compounds (Figure 1B).

**Figure 1:**
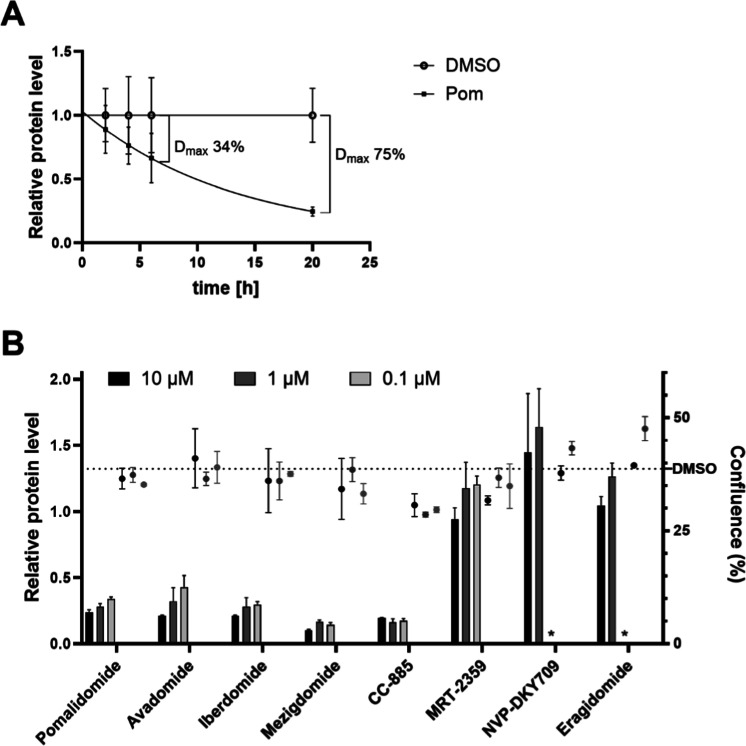
Relative IKZF1 protein level measured by HTRF. (A) Multiple end-point study: Maximal degradation reaches 11, 24, 34, or 75% after 2, 4, 6, or 20 h treatment with 10 μM pomalidomide. (B) Compound profiling: Relative protein level after 20 h treatment with different CELMoDs recapitulates published compound activities (bar graphs plotted on the left *y*-axis, measured by TR-FRET and normalized to the DMSO vehicle). Confluence (%) was measured in parallel to signal potential toxic effects, as may be the case for CC-885 (scatter dots plotted on the right *y*-axis, measured via brightfield microscopy). Data are means of *N* = 3 ± SD, *condition not included.

### Direct End-Point Measurement of the Target Protein Level: Assay Performance in Screening

Aiming to understand the assays’ strength and limitations for screening medium-sized chemical libraries, we designed a validation library (Figure 2, design details in Supporting Information 1 and full composition in Supporting Information 2). Briefly, this library of 941 small molecules was assembled from (i) annotated compounds (∼1/3 of the set) that have extensively been studied both in biochemical and in functional assays, with a well-understood bioactivity and mode of action including known molecular degraders of diverse proteins engaging different E3 ligases (both bifunctionals and glue degraders; “bioactives” in Figure 2) and (ii) frequent hitters (FHs), i.e., compounds with a high likelihood of appearing active in various biological assays without following the sought mechanism (as predicted by the Hit Dexter machine learning model;^10^ ∼1/3 of the set). Finally, this validation library included (iii) a subset of structurally blinded proprietary compounds (∼1/3 of the set) that consisted of small molecules with well-understood specific bioactivities. This latter part of the library was not explicitly enriched in targeted molecular degraders but may have included CELMoDs at low proportion, hence overall reflecting the makeup of the disclosed bioactives subset and serving to test our setup (Table S1 in Supporting Information 1; disclosed compounds SMILES in Supporting Information 2).

**Figure 2:**
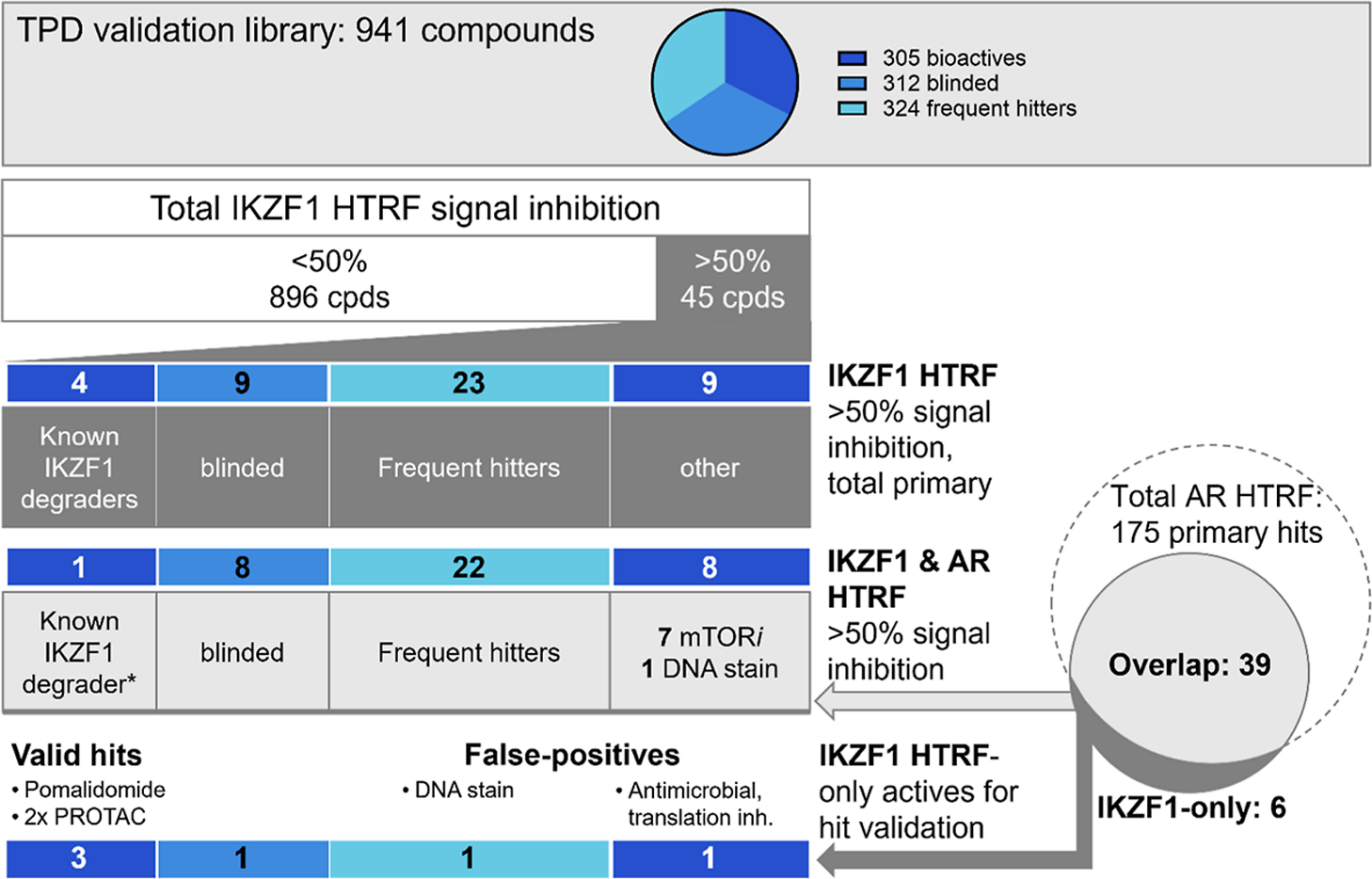
TPD validation library subsets and analysis of primary hits from the IKZF1 HTRF screen. Primary hits are defined as inhibiting the signal by at least 50% after signal normalization (0% inhibition—vehicle and 100% inhibition—pomalidomide (IKZF1) or ARCC-4 (AR)). AR: Androgen receptor.

In the screening mode, the assay achieved acceptable statistical consistency,^21^ and 45 primary hits were identified (Figure 2; *Z*′-values ranged from 0.5 to 0.6). Among these primary hits were pomalidomide as well as three PROTACs that incorporate pomalidomide-derived E3 binders and thus induce degradation of IKZF1 as off-target. After removal of 9 blinded compounds, the remaining 32 primary hits contained 23 FHs according to the cell-based prediction model.^10^ These included inhibitors of topoisomerase, protein synthesis, or ATPases and fluorescent compounds/DNA intercalators. Of the remaining 9 hits, the majority were inhibitors of mTOR that were not flagged by the FH prediction model.

Assuming that the same assay principle, screening workflow, and library applied to an unrelated target may serve as a counter strategy to invalidate hits, we analyzed the overlap of primary hits from the IKZF1 HTRF screen with a similarly executed HTRF screen that measured endogenous androgen receptor (AR) levels. The AR is a transcription factor that plays a central role in androgen-dependent diseases such as prostate cancer. It is hence a major target for degrader development in this context, and our validation library included known targeted degraders of AR. Importantly, AR differs from zinc finger transcription factors including IKZF1 in terms of both structure and native interactome, and no cross-regulation is to be expected between these targets in the studied systems. Further, while the AR screen used a different cell model and measured an unmodified, endogenous target, compound incubation time was kept identical for the AR and the IKZF1 screen, an important factor when analyzing unspecific compound effects. Our analysis identified 39 shared primary hits whereof the majority were predicted FHs^10^ or known to inhibit mTOR (Figure 2). Hence, while the primary hit rate was at 4.8%, the counter assay flagged almost 90% of these hits as unspecific, leaving six for downstream validation. From these, three compounds were confirmed IKZF1 degraders, while the remaining two false-positives included a DNA stain and a translation inhibitor (one compound was blinded). However, this validation strategy would not necessarily have worked the other way round, using the overlap with the IKZF1 screen to remove unspecific compounds from the AR screen, as in the latter case, the primary hit rate was higher (23%).

In summary, we implemented a homogeneous sandwich immunoassay that achieves specific detection and reliable quantification of the target protein directly from the cell lysate, thereby enabling degrader discovery in a screenable format without the need for tagged proteins. While this assay captured known target degraders from the validation library, primary hits were enriched in FHs and included compounds with generalized effects such as translation inhibition/cell cycle arrest. These false-positives were largely invalidated through a similarly executed screen on a different target.

### Degrader-Induced Recovery of Cell Growth: Assay Adaptation and Optimization

An alternative approach to eliminate compounds with generalized effects such as translation inhibition from primary hits in degrader screening is the signal rescue format first introduced by Koduri et al.^9^ This assay couples TPD to a positive readout, specifically restoration of cell growth.^22–25^ The assay principle relies on fast-growing cells that are stably transduced to bicistronically coexpress (i) the target protein fused to a suicide kinase (dCK*) and (ii) eGFP, serving as an expression marker both during cell line generation and degrader screening. The suicide substrate 5-bromovinyl uridine (BVdU) selectively inhibits cell growth in dCK*-positive cells, while dCK*-negative cells remain unaffected. In cells expressing the dCK*-target fusion protein, growth is recovered by a molecular degrader of the target protein due to codegradation of dCK* (Figure 3, top). This way, compounds with described generalized effects would not be identified as hits. A detailed description of the assay principle is provided in Supporting Information 1.

**Figure 3:**
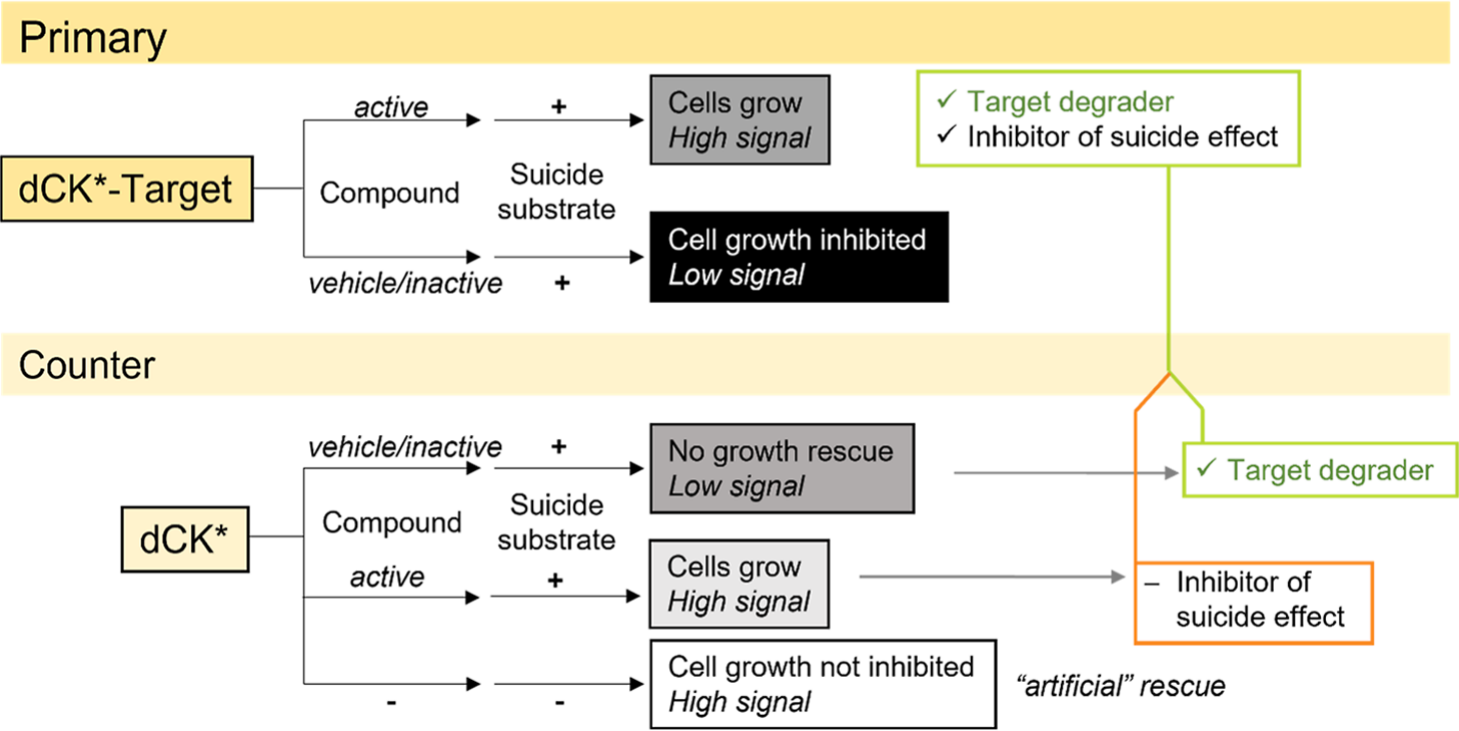
Assay principle of the growth recovery assay. The primary readout of this format measures cell growth at 5 days after treatment initiation, and hence, compounds perturbing essential cellular functions will not be among primary hits. The counter assay invalidates primary hits that target suicide kinase-related components.

As this assay in its originally described version in our hands did not reliably achieve a sufficient assay window and robustness for single-point screening, we extensively optimized the protocol while maintaining its rational basis. Briefly, the GFP-based object counting in our hands underestimated the actual cell number present in the well. This underestimation was especially pronounced at higher cell densities, and consequently, the high control signal was artificially lowered. To improve object separation during image analysis while keeping acquisition times short, we switched the assessment of cell growth to Hoechst nuclear staining-based object counting, which more faithfully reflected cell count and thus increased the assay window by over 3-fold (Figure 4A).

**Figure 4:**
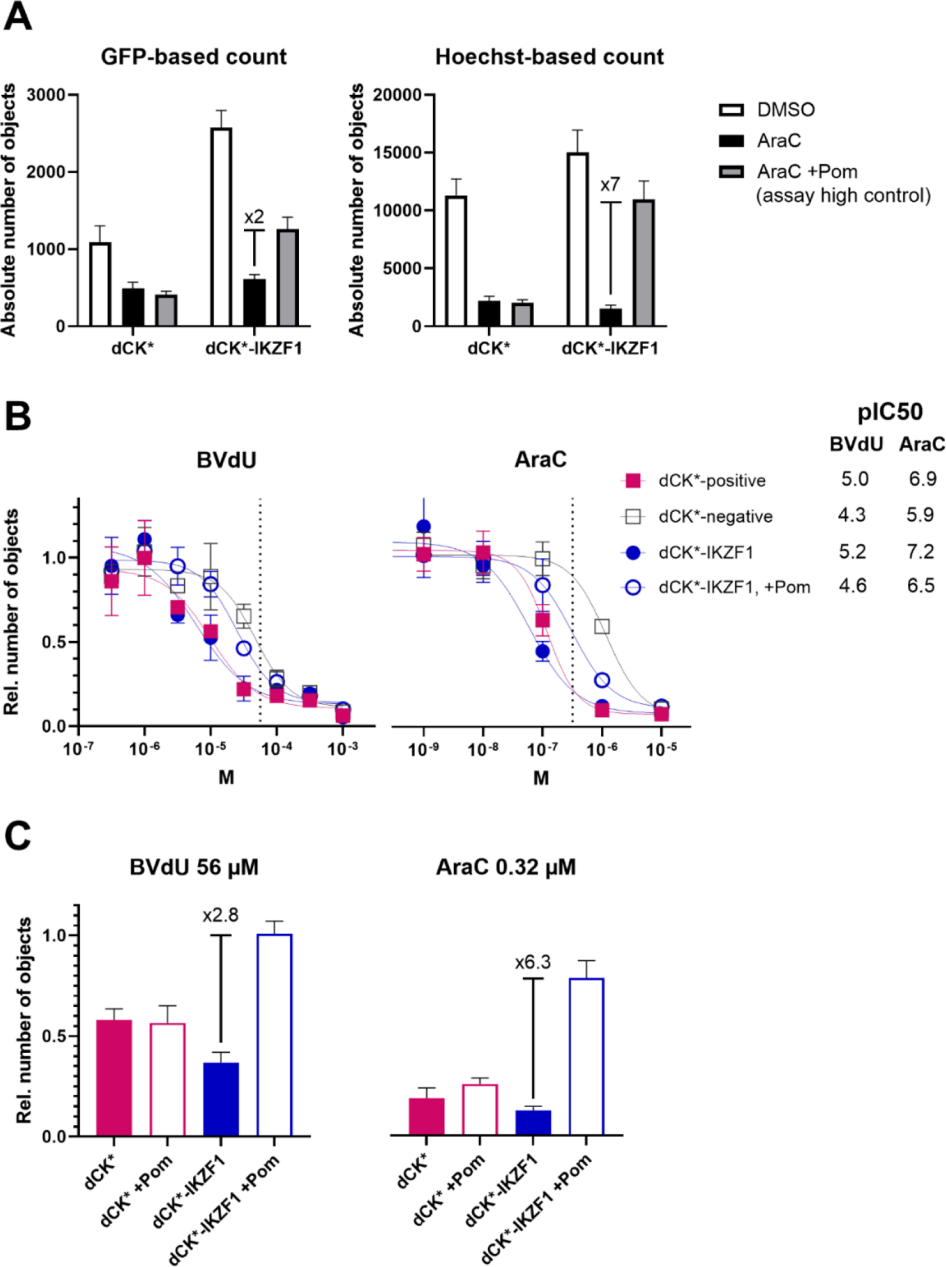
Optimization of the assay window. (A) Comparison of GFP-based versus Hoechst-based object counting. Absolute objects are retrieved from images of whole wells in 384-format before (GFP) or after live-cell nuclear staining (Hoechst 33342) (*N* = 8 ± SD). To compare assay windows, signal-over-background is indicated by x2 and x7, respectively. (B) Concentration-dependent BVdU- or AraC-induced suicide effect in dCK*-positive (■) versus -negative cells (□) and in dCK*-IKZF1 cells pretreated with DMSO (●) versus pomalidomide (○) (*N* = 3 ± SD). Potency of the suicide substrate under each condition is indicated as pIC_50_, meaning the negative log of the IC_50_ value that represents 50% inhibitory concentration. Dotted lines indicate concentrations chosen for the screening assay. (C) Comparison of the suicide substrate effect in the screening layout. Pomalidomide pretreatment shows a significant rescue effect in dCK*-IKZF1 but not in dCK* cells under both suicide substrate conditions, yielding the largest assay window for 0.32 μM AraC as indicated by signal-over-background x6.3 versus x2.8 for the BVdU condition. In (B,C), objects represent nuclei and are retrieved via live-cell Hoechst-staining and image-based counting from whole wells in a 384-well plate format (*N* = 8 ± SD). Note that in concentration–response experiments, both BVdU and AraC appear to have increased potency due to the difference in final DMSO ((B) 1.05% and (C) 0.15%).

Furthermore, we enhanced BVdU potency by cotreating cells with TAS-114, a dual dUTPase/dihydropyrimidine dehydrogenase inhibitor, and achieved an almost 4-fold assay window (Figure S1 in the Supporting Information 1).^26^ Still, aiming for an effective suicide substrate, we finally substituted BVdU with the nucleoside analogue cytarabine (AraC), an antineoplastic antimetabolite.^27^ In concentration–response experiments, we found that AraC was more potent than BVdU (2-log difference in IC_50_-values). Importantly, AraC achieved adequate selectivity, probably due to the overexpression of constitutively active kinase dCK* rather than differences in substrate preference for dCK* versus cellular dCK (Figure 4B).^25^ Finally, in the screening layout, this setup achieved an assay window of 6-fold under 0.32 μM AraC-treatment, which we could not reach with BVdU (Figure 4C).

Next, we assessed the growth recovery assay performance in degrader profiling (Figure 5A). As anticipated, the results from the recovery assay presented a nearly opposite scenario compared to the signal inhibition configuration with IKZF1 degraders giving a high signal. Chemically related compounds that are known to leave IKZF1 levels unaffected yielded low signal and, interestingly, compounds targeting proteins that are essential for cell growth even scored below baseline, e.g., CC-885 (Figure 5B). This effect was more pronounced with longer compound pretreatment, as indicated by comparing relative cell growth under CC-885 treatment overnight or for 48 h, and may assist in interpretation of screening results (Figure 5C, indicated by dashed line).

**Figure 5:**
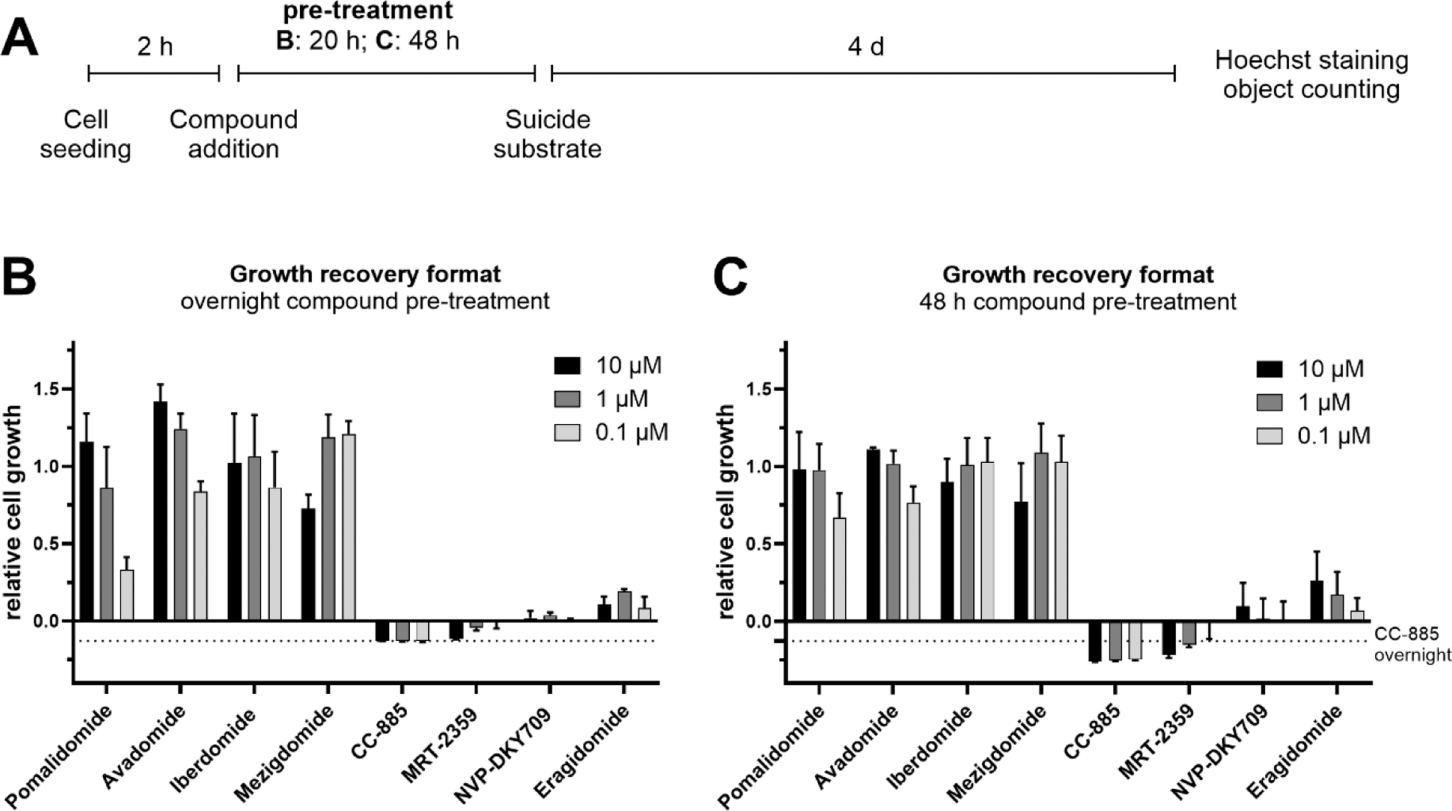
Growth recovery of dCK*-IKZF1-modified cells by different CELMoDs. (A) Consecutive steps and timeline of the growth recovery assay. (B) Relative cell growth determined via Hoechst-based object counting after 20 h compound pretreatment, followed by 4 days of suicide substrate (AraC) treatment (normalized to pomalidomide-treated control wells as full growth recovery and vehicle-treated wells as a negative control). (C) Variation of (A) where the compound pretreatment time was increased to 48 h. To enable comparison of the compound-induced growth inhibitory effect at 20 or 48 h pretreatment, dashed lines in (B,C) indicate the mean relative cell growth in wells treated for 20 h with CC-885 (no difference in effect for the three tested concentrations).

We also explored the transfer of this assay principle to other targets. As this format heavily relies on fast basal growth kinetics, transfer to other cell lines was not attempted. While for TPD-known targets validated degraders represent the prime choice as pharmacological growth rescue control (e.g., pomalidomide for IKZF1), these are not available for novel targets, which thus require a generally applicable and, hence, off-mechanism control. The original reported protocol used dipyridamole, a nucleoside transport inhibitor;^9^ however, this approach was incompatible with our readout as it completely prevented Hoechst staining and, in our hands, increased GFP intensity compared to untreated control, an observation we did not follow up on. By leaving out the suicide substrate, 100% growth rescue can be simulated; it is important to note, however, that even potent degraders may only rescue to ∼50% growth compared to the non-AraC-treated condition, as is the case for the dCK*-IKZF1 cell line–pomalidomide pair. Reasons for this may include the high expression level of the dCK*-target fusion protein. Moreover, blocking the AraC effect via competition with the natural nucleoside 2′-deoxycytidine efficiently rescues cell growth (Figure S2B in Supporting Information 1). With regard to target space, the applicability of this assay format is expected to be limited and may exclude essential targets since growth recovery may not be achieved in such a case (see the example of CC-885, Figure 5B,C). We have successfully transferred the assay principle to Bassoon (BSN) as a potential degrader target, analogously as done for IKZF1, by first generating cells that stably express a fusion protein of dCK* and a relevant section of the BSN protein (“BSN^short^”, as full-length BSN is too large for the lentiviral system used) and next confirming efficient cell growth inhibition by AraC-treatment that is reversed by dC-competition (Figure S2B in Supporting Information 1). Moreover, transfer of the growth inhibition system to RBM39 as a degrader target was successful; however, we were surprised by the finding that indisulam, a previously validated RBM39 degrader, failed to rescue cell growth in this system (Figure S2A in Supporting Information 1). In the orthogonal FRET-based sandwich immunoassay, we found that indisulam indeed does not efficiently induce degradation of the dCK*-RBM39 fusion protein, while potently degrading endogenous RBM39. Possible explanations may include overloading specific UPS components by attempting to induce degradation of a highly expressed ectopic protein or a change in the localization of the modified versus endogenous target, again highlighting some of the limitations of this approach.

### Degrader-Induced Recovery of Cell Growth: Assay Performance in Screening

When applied for screening the same TPD validation library as described for the FRET-based assay, the growth recovery format performed robustly as well (*Z*′-values of 0.4–0.5).^21^ While for the primary assay in dCK*-IKZF1-modified cells, pomalidomide served as on-target growth recovery control, for the counter assay, non-AraC-treated wells served as simulated growth recovery control. Due to these differences in normalization, thresholds varied slightly between primary and counter screen, with hits being defined as rescuing growth in the primary assay by at least ten times the standard deviation of the 0% growth condition (>10×σ_Low_), and no more than 5 times the standard deviation from the 0% growth conditions in the counter assay (<5×σ_Low_, indicated by dashed green lines in Figure 6A). The resulting 7 hits included pomalidomide from within the library and library-PROTACs that incorporate pomalidomide-like E3-binders, hence capturing an off-target effect of CRBN-engaging PROTACs (Figure 6A, green stars). As expected, PROTACs engaging other E3-ligases and, importantly, those targeting CRBN through advanced thalidomide 5-fluoride-derived binders (e.g., ARV-110) were not identified as hits. This was confirmed in concentration–response experiments (data not shown).

**Figure 6:**
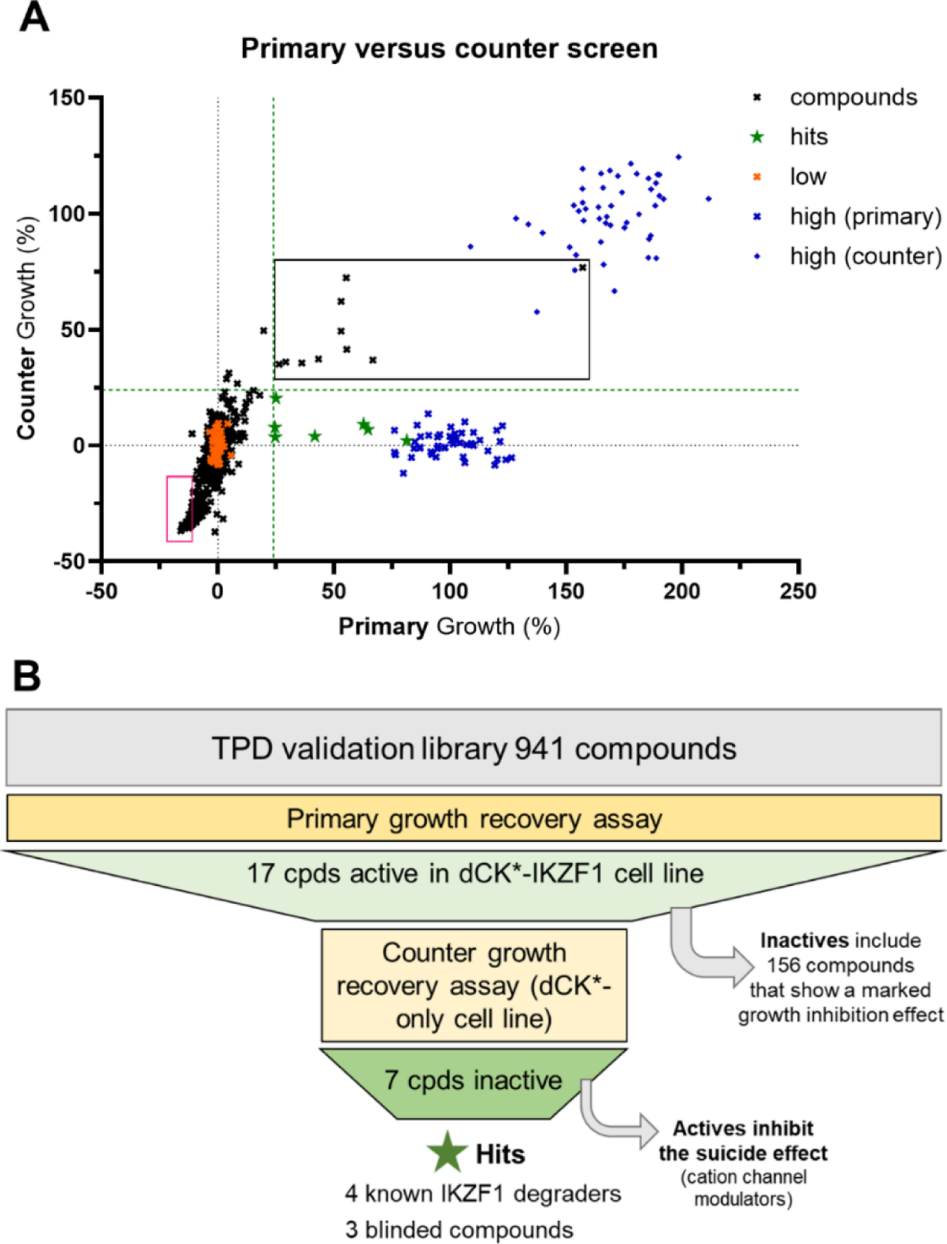
TPD validation library screening in the growth recovery assay. (A) Relative growth recovery by library compounds or controls in primary versus counter assay. Primary assay: dCK*-IKZF1-modified cells, growth recovery achieved by 10 μM pomalidomide is defined as 100% (*x*-axis); counter assay: dCK*-modified cells, “no suicide”-control as 100% growth (simulated recovery, *y*-axis). Compounds from the TPD validation library were screened at 5 μM, and primary hits are defined as recovering growth by at least ten times the standard deviation of the vehicle-treated control (low) over the low mean (indicated by the green dashed line on the *x*-axis). Hits from the primary screen that were not invalidated by the counter screen are highlighted as green stars while those that were also active in the counter screen are within the black rectangle (here, the limit was set at five times the standard deviation of the vehicle-treated control (low) over the low mean, which is indicated by the green dashed line on the *y*-axis). Compounds within the section in magenta show a marked growth inhibitory effect in both primary and counter screen, which is explained by an inhibition of the suicide effect. (B) Schematic overview of the compound-focused analysis of results from the growth recovery primary versus counter screen.

To gain a detailed understanding of the strengths and limitations also of this format, we analyzed those hits from the primary screen that were invalidated by the counter assay (total of 10 compounds; Figure 6B). Among these compounds, cation channel modulators were present (lidoflazine, FPL64176, SR1078, and A-784168). In line with the compounds’ signal rescue effects in dCK*-only cells, the variety of agonist/antagonist modulation and the structural diversity found in these compounds suggests a higher probability of target-unrelated cellular effects (ultimately leading to an inhibition of the suicide effect) rather than selective modulation of IKZF1-related cellular mechanisms. Accordingly, the majority of these false-positives were also active when a different target was screened in this format (not shown).

Importantly, a significant proportion of the library compounds elicited substantial growth inhibitory effects on top of the AraC-induced inhibition, both in the primary and counter assay (Figure 6, magenta). This effect had also been noted for some of the CELMoD’s described above, most prominently CC-885. With the aim to potentially exploit this “multiplexing” of growth inhibitory and recovery effects, we performed a detailed analysis of the inhibitory compounds (Figure 6B and Table S2). Among the 156 compounds that yielded below -11% growth, 28 structures were blinded (i.e., ∼10% of the blinded subset elicited this inhibitory effect). Out of the remaining 128 nonblinded compounds, only 25 were from the disclosed bioactives subset, confirming that ∼10% of nonpromiscuous bioactives elicit an inhibitory effect in this assay. These included assay principle-related inhibitors (cyclocytidine: a prodrug form of AraC, probably enhancing the AraC effect by increasing its final concentration; decitabine: a DNA-methyltransferase inhibitor that requires activation by dCK) and inhibitors of central cellular nodes (topoisomerase, PI3K/mTOR, and CDK). As expected, from the FH^10^ subset of the library, we find growth inhibitory compounds at an increased rate of ∼30% (103 compounds). These included members from the anthracycline class or polycyclic quinoids (e.g., epirubicin and mitoxantrone), other topoisomerase inhibitors, as well as inhibitors of protein synthesis.

### Orthogonal Degrader Assays in a Screening Cascade

Finally, we performed another small-scale screen of a different compound set with a similar size (1001 small molecules, SMILES in Supporting Information 2). Molecules were selected based on structural features known from the few well-established MGDs (e.g., aryl sulfonamides, phthalimides, pyridyl thiazole amines, triazino indoles, etc.). As a first, strong, and cost-efficient filter, we employed the growth recovery assay (primary and counter) to then use the FRET-based format for hit profiling. The growth recovery screening in dCK*-IKZF1 versus dCK*-only cells resulted in 9 hits (0.9% hit rate; Figure 7A,B, green stars). While the majority of compounds in the set do not have related bioassay annotation data, some were known thalidomide analogues with reported activities in relevant assays. For example, 4- and 5-hydroxythalidomide have been reported inactive toward IKZF1-degradation in a structure–activity relationship study while still binding CRBN,^19^ and in accordance with reports, these compounds were not among hits in the screen, indicating the suitability of our assay as a source of TPD-mediated primary hits.

**Figure 7:**
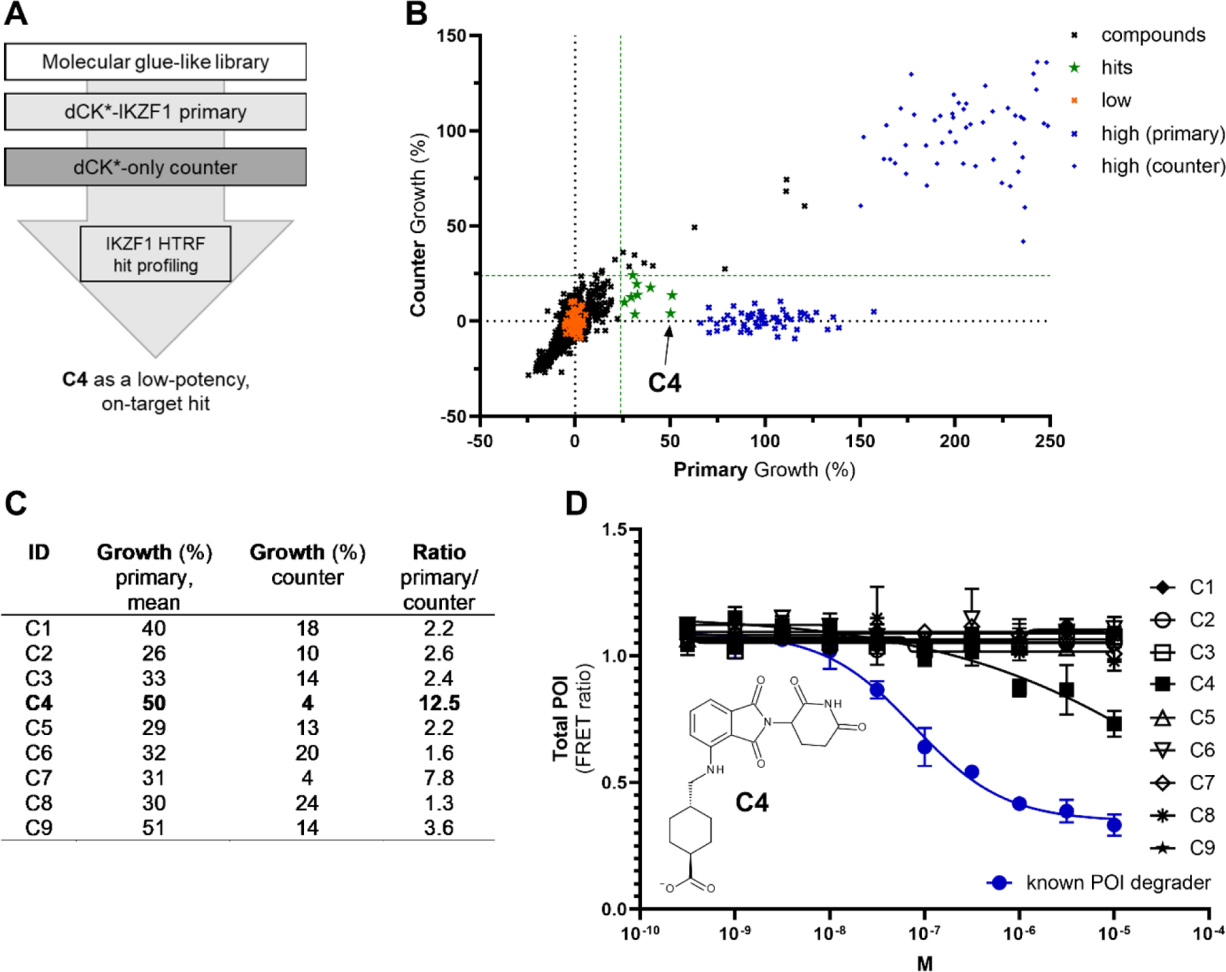
Proof-of-concept study. (A) Overview of the screening flow. (B) Relative growth recovery by compounds or controls in primary (dCK*-IKZF1; *x*-axis) versus counter (dCK*-only) setup (*y*-axis). Library compounds (1001 small molecules) were screened at 5 μM. Nine hits were identified (green stars; as before, the limits were set at recovering growth to at least ten times the standard deviation of the vehicle-treated control (low) over the low mean in primary and no more than five times the standard deviation of the vehicle-treated control (low) over the low mean in the counter assay, indicated by the green dashed lines). (C) Table showing growth (%)-values from primary (*N* = 2) and counter as well as the calculated selectivity ratio for the 9 hit compounds. (D) Profiling of the 9 hits with the FRET-based assay in triplicate 10-point half-log concentration–response and in direct comparison with pomalidomide as a known IKZF1 degrader; molecular structure of C4.

Next, all 9 hits were profiled in triplicate 10-point half-log concentration–response curves using the FRET-based assay (Figure 7D). Importantly, general inhibitory effects were already excluded as all 9 compounds had passed the signal rescue filters, where, as established above, growth recovery cannot be achieved with compounds exhibiting, e.g., general inhibition of protein synthesis. False-positives from the primary screen may include compounds that interfere with the expression of the integrated exogenous gene more specifically as well as those leading to degradation of the fusion protein not via IKZF1 but via dCK*. These compounds, however, would also be active in the dCK*-only counter screen and were thus already largely invalidated. Still, as in any screen, the hit criteria may not perfectly filter for bona fide degraders of the target only and may thus still include off-target, off-mechanism chemistry. Data-driven fine-tuning of the selection criteria may further enhance efficiency by increasing the true positive to false positive ratio. In this study, the aim was to allow for the inclusion of weak (or slow) target degraders, which may also mean to include some off-target chemistry, while efficiently filtering out generalized effect compounds and FHs that may be tough to invalidate in later cascades. The most potent and selective of the 9 hit compounds (compound C4 in Figure 7C) showed concentration-dependent inhibition of FRET ratio, indicating a likewise decrease in target protein levels. This validated hit compound C4 incorporates the full phthalimide structure of pomalidomide, featuring a PROTAC-linker-like attachment at the 4-amino position. 4-Amino-substitution in pomalidomide has extensively been studied with regard to neosubstrate degradation, and it has been demonstrated that when preserving the C4-amino group for hydrogen bonding, a productive ternary complex with IKZF1 is formed. Still, while incorporating the same chemical core structure, C4 is less potent than pomalidomide, potentially due to its carboxylate function that may limit cell permeability (*c* Log *D*(pomalidomide) = 2.2; *c* Log *D*(C4) = 1.3); in fact, C4 might have been overlooked in a direct FRET-based screen (note that here cells are being treated overnight while treatment duration is 5 days in the growth recovery assay). It is generally desired to efficiently identify even low-potency but “on-mechanism” hits since after screening (in hit-to-lead development), medicinal chemistry strategies can increase potency of otherwise favorable compounds (e.g., regarding the “on-mechanism” feature and scaffold novelty). A scenario where primary screening provides less efficient hit filtering may not allow setting the hit threshold at levels that include these low-potency, “true positive” hits, simply because the high number of hits implies a high proportion of false-positives and necessitates extensive further hit validation. As a consequence, promising chemical starting points may be missed (false-negatives).

## Conclusions

Others have highlighted the significant challenges in designing and interpreting cascade experiments for identifying, triaging, and validating hits from a degrader screening campaign, ultimately aiming to identify bona fide molecular degraders,^6,8^ and it appears highly desirable to incorporate high-fidelity assays with interpretable outcomes early in these screening workflows. We first rigorously addressed critical parameters for the implementation of degrader assays for screening. Second, our validation library was specifically designed to analyze hit populations arising from different TPD assays used for screening to ultimately enable faithful interpretation of screening results. This allowed the side-by-side evaluation of two complementary assay formats regarding performance and hit populations, revealing their individual strengths and limitations and thus guiding the design of a proof-of-concept study. Here, compound C4 was identified as a bona fide, although low-potency, degrader of the transcription factor IKZF1. In conclusion, our results clearly illustrate that the cascade and filters applied are highly robust and sensitive to efficiently identify even low-potency on-target and on-mechanism chemistry. Further work including different targets and a larger screening set is needed to provide an estimation of the general applicability of this approach, and during hit validation, compound triaging may benefit from consideration of degradation kinetics.

## Methods

Suppliers and catalogue numbers for all commercial materials used in this study are provided in Supporting Information 1.

### Cell Lines and Culture

HEK293FT cells were grown according to manufacturer’s specifications in a complete medium consisting of Dulbecco’s modified Eagle’s medium (high glucose) supplemented with 10% FBS, 6 mM l-glutamine, 1% penicillin/streptomycin, 1× MEM nonessential amino acids, and 1 mM sodium pyruvate at 37 °C and 5% CO_2_. To maintain growing cultures of unmodified HEK293FT cells, the complete medium was supplemented with 500 μg/mL G-418, and for growing cultures of stably transduced, HEK293FT-derived cell lines, 8 μg/mL Blasticidin was added on top of this. During assays, no selection antibiotics were present.

For details on generation of stably transduced cell lines, see Supporting Information 1.

### Compounds Used in This Study

The small-molecule library we used as a platform for initial screening (validation library composition in Supporting Information 2) is composed of 941 compounds, partly derived from in-house drug repurposing collections,^28^ where a curated database is publicly available listing the compounds, indications, primary targets (where known), and mechanism of action.^29^ Additionally, known molecular degraders (Molport) and structurally blinded compounds from a well-annotated Chemical Biology library were included. A third subset consisted of predicted FHs (identified from in-house small-molecule libraries via an ML prediction algorithm for FHs, Hit Dexter 2;^10^ details in Supporting Information 1).

The small-molecule library used to test our optimized assay setup is composed of 1001 compounds that were selected based on scaffold similarity to published MGDs (Specs). Scaffolds present in the set include aryl sulfonamides, pyridyl thiazole amines, triazino indoles, and phthalimides. All known compounds structures are disclosed in Supporting Information 2.

An analysis of physicochemical properties of the two screening libraries is provided in Figure S3 in Supporting Information 1.

Other small-molecule compounds used in this study were commercially sourced (vendor and catalog no. in Supporting Information 1).

### Homogeneous Sandwich Immunoassay

#### General Procedure

The assay was performed using the respective assay kit including lysis buffer, labeled antibodies, and diluents according to the manual and as specified below (Revvity; IKZF1: HTRF Human and Mouse Total IKZF1 Detection Kit 64IKZF1TPEG; AR: HTRF Human Androgen Receptor Detection Kit 64ANDRPEG; RBM39: HTRF Human and Mouse Total RBM39 Detection Kit 64RBM39TPEG).

Briefly, stably transduced dCK*-IKZF1-HEK293FT cells were seeded in 20 μL of complete medium per well in 384-well plates (SpectraPlate-384, Revvity, 6007650) and allowed to adhere overnight at 37 °C and 5% CO_2_ before the compound from the DMSO stock or vehicle was added using an Echo acoustic dispenser (Labcyte). Plates were incubated overnight at 37 °C and 5% CO_2_. Next, the treatment medium was slowly aspirated from all wells using a Janus MDT liquid handling station equipped with 384-well robotic tips (Axygen, Corning, PK-384-R). Lysis buffer was dispensed manually (20 μL per well). Plates were briefly centrifuged and incubated with a lid at room temperature for 30 min. The detection antibody mix (3 μL/well) was manually dispensed into detection plates (ProxiPlate 384-shallow well Plus, Revvity, 6008280). Next, using the Janus MDT station equipped as above, cell lysates were homogenized by carefully pipetting up and down and lysates transferred to the prefilled detection plate (12 μL/well). Plates were sealed and incubated overnight before the TR-FRET signal was read on an EnVision multimode reader (Revvity; Flash lamp, 100 flashes; excitation: 320 nm, bandwidth 75 nm; emission: channel 1620 nm, bandwidth 10 nm; channel 2665 nm, bandwidth 7.5 nm; and delay 60 ms). FRET ratio was calculated as channel 2/channel 1, and background-subtracted data were normalized to vehicle (DMSO, 0% inhibition) and positive control (pomalidomide, 100% inhibition). Wells for background did not receive cells but included complete medium, DMSO-treatment, equivalent workup, and antibody mix.

#### Concentration–Response Experiments

To evaluate the inhibitory effect of compound treatment on the dCK*-IKZF1 protein level, quantified via the FRET ratio, 2000 dCK*-IKZF1 cells/well were seeded according to the general procedure, and 10-point half-log serial dilutions starting from 10 μM (20 nL from 10 mM DMSO stock) were added the next day. Per plate, at least 8 pomalidomide-treated (10 μM) and at least 8 DMSO-treated wells were included for normalization.

#### Screening

According to the general procedure, 2000 cells/well were seeded in columns 2–24, and column 1 received complete medium. Library compounds were dispensed into single wells at a final concentration of 5 μM (10 nL from 10 mM DMSO stock) in plate columns 3–22. Columns 1 and 24 received DMSO (10 nL), and column 23 received 10 μM pomalidomide (10 nL from 20 mM DMSO stock).

### Growth Recovery Assay

#### General Protocol

Stably modified HEK293FT cells were seeded in 20 μL of complete medium at a density of 300 cells/well in 384-well imaging plates (PhenoPlate 384-well; Revvity 6057302) using a Multidrop Combi + dispenser (Thermo Fisher Scientific) equipped with a standard cassette (Cat. no. 24072670). At 2 h after seeding, compounds or vehicle were added using an Echo acoustic dispenser (step 1). Plates were incubated overnight at 37 °C and 5% CO_2_ before suicide substrate was added via acoustic transfer (step 2). To allow for optimal cell growth during the following 4 days, 20 μL of complete medium was added using a Multidrop Combi + dispenser, and plates were again incubated at 37 °C and 5% CO_2_. At day 5 post cell seeding, 10 mg/mL Hoechst 33342 in dd water was diluted 1:1000 in PBS, and all wells received 10 μL per well of this dilution using a Multidrop Combi + dispenser (final Hoechst concentration 2 μg/mL). Plates were incubated for 45 min at 37 °C and 5% CO_2_ before being imaged (EnSight multimode plate reader, Revvity; inverted optical microscope with 4× magnification and sCMOS image sensor). Objects were detected in full wells (BLUE channel, excitation 385 nm, 30 ms, 100%) and counted (Kaleido 3.0, Object detection method C).

#### Concentration–Response Experiments: Suicide Substrate

To evaluate the potency of 5-bromovinyl-deoxyuridine (BVdU) and cytarabine (AraC), 10-point half-log dilutions (BVdU) or log-dilutions (AraC) starting from 0.1 M were prepared manually in DMSO. The assay was performed according to the general protocol described above using, in step 1, 10 mM pomalidomide (20 nL of 10 mM DMSO stock) as a signal rescue control for dCK*-IKZF1 cells and vehicle (DMSO) for all other conditions. On the next day, 400 nL of suicide substrate dilutions was dispensed in quadruplicate (step 2), and the general protocol was followed (final concentration ranges: BVdU 1 mM–30 nM, AraC 1 mM–1 pM; final DMSO 1.05%). For all cell lines, normalization was based on the object count averaged from at least 8 wells that received vehicle only (100% growth).

#### Concentration–Response Experiments: Degraders

To evaluate the rescue effect that different IKZF1-degraders have on dCK*-IKZF1 cells under suicide substrate-induced growth inhibition, 10-point half-log serial dilutions of the degraders starting from 10 mM were prepared manually in DMSO and 20 nL of these dilutions were dispensed in quadruplicate during step 1 of the general protocol described above. In step 2, AraC was added to all wells at a final concentration of 0.3 μM (40 nL from 0.3 mM DMSO stock), and the general protocol was followed (final DMSO 0.15%).

#### Screening

In step 1 according to the general protocol, library compounds were dispensed into single wells at a final concentration of 5 μM (10 nL from 10 mM DMSO stock) in plate columns 3–22. Columns 2 and 24 received 10 μM pomalidomide (10 nL from 20 mM DMSO stock), and columns 1 and 23 received DMSO only (10 nL). In step 2, columns 3–24 received AraC (40 nL of 0.3 mM) and columns 1 and 2 received DMSO only (40 nL).

### Statistical Analysis

#### Primary Growth Recovery Screening

From Hoechst-based object count for each well growth (%) was determined by normalization on a plate basis to pomalidomide-treated wells (10 μM, *n* = 16, 100% growth) and vehicle-treated wells (DMSO, *n* = 16, 0% growth). Each compound was tested in duplicate at 5 μM on different screening days, and mean growth (%) was calculated. Outliers in positive and negative control wells were invalidated when exceeding 3× standard deviation of the mean to increase robustness and reliability.

#### Counter Screening for Growth Recovery

Growth (%) was determined from the Hoechst-based object count by normalization on a plate basis to vehicle-treated wells that did not receive cytarabine (*n* = 16, 100% growth) and vehicle-treated wells that did receive cytarabine (DMSO, *n* = 16, 0% growth). Each drug was tested in singlicate at 5 μM. Outliers in positive and negative control wells were invalidated when exceeding the 3× standard deviation of mean to increase robustness and reliability.

#### Hit Detection in Growth Recovery Format

Due to the differences in normalization for primary versus counter (simulated 100% rescue), we set different thresholds for hit detection: hits were defined as yielding signals above 10 × SD_Low_ over low control in the primary (dCK*-IKZF1) and below 5 × SD_Low_ over low control in the counter assay (dCK*), SD_Low_ = standard deviation of the low control.

#### FRET-Based Screening

Inhibition (%) was determined from FRET ratio by normalization on a plate basis to pomalidomide-treated wells (10 μM, *n* = 16, 100% inhibition) and vehicle-treated wells (DMSO, *n* = 16, 0% inhibition). Outliers in positive and negative control wells were invalidated when exceeding 3× standard deviation of the mean to increase robustness and reliability.

Data were analyzed using IDBS Activity Base, KNIME, and plotted using Spotfire, GraphPad Prism v10.

#### Concentration–Response Experiments

For determining half-maximal inhibitory concentration (IC_50_) values for BVdU and AraC, the Hoechst-based object count from wells treated with log- or half-log serial compound dilutions was normalized to averaged object counts from at least 8 wells that received DMSO vehicle instead of the suicide substrate (100% growth).

For determining IC_50_-values for hits from screening and additional CELMoDs in the signal rescue or signal inhibition format, each condition was tested in triplicate, and normalization was done as described for screening.

Data were analyzed and plotted using GraphPad Prism v10, and IC_50_ values were determined using the “[inhibitor] versus response, variable slope (four parameters)” analysis mode.

## Supplementary Material





## Notes

Growth recovery assay principle, optimization, and transfer to IKZF1-unrelated targets; physicochemical properties of screening libraries; library composition and statistical hit analysis; and commercial material vendor catalogue numbers, plasmids, and cell line generation (PDF)
Screening data and composition of libraries including SMILES (nonblinded compounds) (XLSX)
